# Transcriptome and Small RNA Sequencing Reveal the Mechanisms Regulating Harvest Index in *Brassica napus*

**DOI:** 10.3389/fpls.2022.855486

**Published:** 2022-04-04

**Authors:** Chao Zhang, Wei Chang, Xiaodong Li, Bo Yang, Liyuan Zhang, Zhongchun Xiao, Jiana Li, Kun Lu

**Affiliations:** ^1^Chongqing Rapeseed Engineering Research Center, College of Agronomy and Biotechnology, Southwest University, Chongqing, China; ^2^Academy of Agricultural Sciences, Southwest University, Chongqing, China; ^3^Oil Research Institute of Guizhou Province, Guizhou Academy of Agricultural Sciences, Guiyang, China; ^4^Engineering Research Center of South Upland Agriculture, Ministry of Education, Chongqing, China

**Keywords:** harvest index, transcriptome, miRNA, regulatory network, *Brassica napus*

## Abstract

Harvest index (HI), the ratio of harvested seed weight to total aboveground biomass weight, is an economically critical value reflecting the convergence of complex agronomic traits. HI values in rapeseed (*Brassica napus*) remain much lower than in other major crops, and the underlying regulatory network is largely unknown. In this study, we performed mRNA and small RNA sequencing to reveal the mechanisms shaping HI in *B. napus* during the seed-filling stage. A total of 8,410 differentially expressed genes (DEGs) between high-HI and low-HI accessions in four tissues (silique pericarp, seed, leaves, and stem) were identified. Combining with co-expression network, 72 gene modules were identified, and a key gene *BnaSTY46* was found to participate in retarded establishment of photosynthetic capacity to influence HI. Further research found that the genes involved in circadian rhythms and response to stimulus may play important roles in HI and that their transcript levels were modulated by differentially expressed microRNAs (DEMs), and we identified 903 microRNAs (miRNAs), including 46 known miRNAs and 857 novel miRNAs. Furthermore, transporter activity-related genes were critical to enhancing HI in good cultivation environments. Of 903 miRNAs, we found that the bna-miR396–Bna.A06SRp34a/Bna.A01EMB3119 pair may control the seed development and the accumulation of storage compounds, thus contributing to higher HI. Our findings uncovered the underlying complex regulatory network behind HI and offer potential approaches to rapeseed improvement.

## Introduction

Harvest index (HI), formerly known as coefficient of economics, refers to the ratio of the economic yield to the biological yield of the crop at harvest (Donald, 1962), which can also be understood as the ratio of seeds harvested from a plant to all the above-ground biomass produced. HI thus reflects the distribution of crop assimilates products in economic yield organs and vegetative organs, and also indicates the patency of crop photosynthate transport from “source” organs to “sink” organs (Bennett et al., 2011, 2012), and HI has clear agronomic implications. Since the first Green Revolution, the increase in the yield of major crops such as rice (*Oryza sativa*), wheat (*Triticum aestivum*), and barley (*Hordeum vulgare*) has been mainly due to the increase in the HI (Austin et al., 1980). The allotetraploid crop rapeseed (*Brassica napus*) is cultivated worldwide for oil, which is extracted from its oil-rich seeds. Luo et al. (2015) examined the *B. napus* genetic architecture of HI using 35,791 high-throughput single nucleotide polymorphisms (SNPs) genotyped by the Illumina Brassica SNP60 Bead Chip in an association panel with 155 accessions, and a total of nine SNPs on the C genome were identified to be significantly associated with HI; they explained 3.42% of the phenotypic variance in HI. Based on Brassica SNP60 Bead Chip, a natural population (NP) containing 520 materials was used to perform genome-wide association study (GWAS) for traits related to HI. Combined with transcriptomic sequencing (RNA-seq) of materials with high and low HI, candidate genes involved in photosynthesis, inflorescence, and silique development were identified (Lu et al., 2016). In addition, in *B. napus*, it was also found that the *BnaA02.TB1* (*BnaA02g14010D*) that regulates lateral branch development and the *BnaA02.GW2* (*BnaA02g18890D*) that regulates grain weight may be related to HI (Chao et al., 2019); *BnaDwf.C9* (*BnaC09g20450D*) and *BnaC04.BIL1* (*BnaC04g41660D*) can affect HI by regulating plant height (Wang X. D. et al., 2020; Yang et al., 2021). These previous studies are of great worth for helping us to elucidate the genetic mechanism of HI in *B. napus*. As seed yield is part of the HI numerator, HI increases with grain yield and has, therefore, received widespread attention in breeding programs (Hay, 1995; Sadras and Lawson, 2011; Beche et al., 2014). Unfortunately, the HI of rapeseed remains much lower than that of other major crops, such as rice, wheat, maize (*Zea mays*), and soybean (*Glycine max*) (Hay, 1995; Unkovich et al., 2010). The HI for rapeseed ranges from 0.05 to 0.42 (Lu et al., 2016), which is far below the theoretical biological limit for HI of ∼0.60 in grain crops (Foulkes et al., 2009), indicating the potential to further increase HI. Therefore, improving HI in rapeseed varieties is a major objective for breeders.

Green plant tissues such as leaves and silique pericarps (SP) constitute “source” organs that are photosynthetically active and provide photoassimilates to “sink” organs like grains and seeds *via* translocation (designated here as “flow”); HI reflects flow and the balance between source tissues and sink organs (Unkovich et al., 2010). In *B. napus*, flow is thought to be the limiting factor in the accumulation of assimilates in seeds (Shen et al., 2010; Fu and Zhou, 2013; Luo et al., 2015). Unlike many other crops in which leaves provide the main source tissue throughout seed development, in *B. napus*, SP take over the role of source tissue from senescent leaves, providing nutrients to sustain the growth of the seed after silique formation (Diepenbrock, 2000; Bennett et al., 2011). Several critical sugar transporter gene families have been identified in *B. napus*, such as *SUCROSE TRANSPORTER* (*BnSUC*), *SUGARS WILL EVENTUALLY BE EXPORTED TRANSPORTER* (*BnSWEET*), and *MONOSACCHARIDE TRANSPORTER* (*BnMST*); transporters encoded by these genes may influence HI positively during the development of siliques by supplying more sugars to the seeds (Jian et al., 2016; Zhang et al., 2020). In addition, the *Arabidopsis thaliana AMINO ACID PERMEASE 2* (*AAP2*) gene encodes an amino acid co-transporter that transports amino acids to the embryo *via* the seed pod vascular system during the seed-filling phase (Hirner et al., 1998), suggesting that SP may supply not only sugars but also other compounds. However, the underlying molecular regulatory mechanism behind the translocation of photoassimilates and other nutrients during the seed-filling phase is poorly understood in *B. napus*.

Harvest index reflects the relative allocation of resources to vegetative and reproductive organs and can be influenced by various environmental factors, including water supply (Gajića et al., 2018), temperature stress (Prasad et al., 2006), and nitrogen (N) fertilization (Amanullah and Shah, 2010). For crop species such as maize, shortening the vegetative growth period reduces the accumulation of photosynthetic products in vegetative organs and raises HI (Hütsch and Schubert, 2017). One method employed to modulate the length of the vegetative period relies on the genetic manipulation of photoperiod sensitivity (Li et al., 2020). In addition, enhancing crop adaptability to external stimuli can also result in significant increases in harvestable products and thus contribute to a higher HI. For example, the rice basic leucine zipper transcription factor bZIP58 induces the expression of seed storage protein genes and starch biosynthetic genes, but its transcripts also undergo alternative splicing in response to heat stress. However, more heat-tolerant rice varieties showed limited alternative splicing and higher expression of seed storage protein genes under heat stress, thus contributing to heat tolerance during grain filling (Xu et al., 2020). In rice, a dominant mutation in the *DENSE AND ERECT PANICLE 1* (*DEP1*) gene displayed insensitivity to N during vegetative growth, which increased N uptake and assimilation and thus improved HI and grain yield at moderate levels of N fertilization (Sun et al., 2014). However, which genes regulate HI by promoting reproductive growth and mediating responses to external stimuli remain largely unknown in *B. napus*.

MicroRNAs (miRNAs) are 20–24-nt single-stranded non-coding RNA molecules that act as key post-transcriptional regulators of gene expression (Voinnet, 2009; Budak and Akpinar, 2015). Extensive studies have revealed that miRNAs play diverse roles during plant development and adjust complex traits in crops (D’Ario et al., 2017; Tang and Chu, 2017). For example, Arabidopsis miR159 lowers the transcript levels of the *MYB DOMAIN PROTEIN 33* (*MYB33*) gene, whose encoded transcription factor normally negatively regulates miR156, thereby modulating vegetative phase change (Guo et al., 2017). Similarly, miR164 induces cleavage of *NAM*, *ATAF1/2*, and *CUC2* (*NAC2*) gene transcripts, which act as negative regulators of drought tolerance in rice (Fang et al., 2014). In Arabidopsis, miR397b regulates *LACCASE 4* (*LAC4*) and influences silique numbers and silique length (Wang et al., 2014). Moreover, rice miR396d forms a molecular bridge between *BRASSINAZOLE-RESISTANT 1* (*BZR1*) and *GROWTH REGULATING FACTORs* (*GRFs*); *OsBZR1* induces the expression of miR396d, which, in turn, represses *OsGRF* transcript accumulation to modulate plant architecture and grain yield (Tang et al., 2018).

The advent of next-generation sequencing technologies has paved the way to cost-effective and highly efficient methods to identify miRNA–mRNA regulatory networks related to complex traits in crops. For instance, embryo development in peanut (*Arachis hypogaea*) is highly sensitive to calcium deficiency in the soil, but the cause for the resulting embryo abortion was unknown. However, an integrated miRNA and mRNA profiling (RNA-seq) analysis revealed that a number of miRNA-mediated regulatory networks affecting seed/embryo development, cell division, cell proliferation, and plant hormone signaling all participated in peanut embryo abortion under calcium deficiency (Chen Z. Y. et al., 2019). Joint RNA-Seq and miRNA profiling analyses further established that miRNAs involved in nitrogen-related pathways regulated the thickness of a pod canopy in *B. napus* (Chen Z. Y. et al., 2019). To date, limited information is available pertaining to miRNA-mediated regulatory networks related to HI.

To better understand the regulatory networks underlying the establishment of HI in *B. napus* during the seed-filling phase, we determined the mRNA and miRNA transcriptome landscape in SP and seeds during the seed-filling stage in plants grown at two locations, Chongqing and Yunnan. We identified potential gene clusters involved in the regulation of HI, with predicted roles in transporter activity and responses to environmental signals. Furthermore, we discovered several miRNA-mediated regulatory networks in SP and seeds. These results contribute to uncovering the complex regulatory networks behind HI and offering potential solutions for its improvement *via* genetic engineering or crop breeding.

## Materials and Methods

### Plant Materials and Field Trials

The *B. napus* accessions YC24 (SWU47), YC52 (Zhongshuang11), and YC46 (Ningyou12) with stability HI for two consecutive years (2012–13 and 2013–14) were selected from 520 accessions in previous study (Lu et al., 2016) and grown in a randomized block design with three replications at Chongqing Beibei (CQ, 29°45′ N, 106°22′ E, 238.6-m altitude) and Yunnan Lincang (YN, 23°43′ N, 100°02′ E, 1819.5-m altitude) during the 2015–2016 growing season. For simplicity, we denoted the planting location first (CQ or YN), followed by the accession number (24, 52, or 46) to distinguish experimental groups. Planting conditions were as previously described (Lu et al., 2016). Plant materials used for RNA-seq and small RNA-seq of growing status are shown in Supplementary Figure 1. We collected HI-related phenotypic traits, such as HI, biomass yield per plant (BY), seed yield per plant (SY), stem dry weight (ST), canopy biomass yield (CBY), according to the calculation method used previously (Lu et al., 2017), and the HI, BY, and SY of CQ24 and CQ46 at 2016 had been shown in our previous study (Zhang et al., 2020).

### RNA Isolation and Library Preparation

For each accession grown at CQ and YN, we harvested seeds (ZS) and SP from the main inflorescence at 30 days after flowering (denoted as 30ZS and 30SP, respectively); there are also leaves (Le, main leaves at 30 days after flowering) and stems (St, main steam at 30 days after flowering) of the same period. For each sample, we collected two biological replicates, each harvested from five independent plants.

Total RNA of each sample was extracted using a CTAB method (Lu et al., 2008). RNA degradation and DNA contamination were monitored by gel electrophoresis on 1% agarose gels. RNA purity was confirmed on a NanoPhotometer spectrophotometer (IMPLEN, CA, United States), and RNA concentration was measured using the Qubit RNA Assay Kit with a Qubit 2.0 Fluorometer (Life Technologies, CA, United States). RNA integrity was assessed using the RNA 6000 Nano Assay Kit on an Agilent Bioanalyzer 2100 system (Technologies, CA, United States). After quality-control, we sent 48 RNA samples for mRNA sequencing and selected 12 samples (including 30SP, 30ZS) for small RNA sequencing to Novogene Corporation (Beijing, China) for library construction and sequencing, as previously described (Chen H. et al., 2019).

### Identification of Differentially Expressed Genes

We evaluated the quality of RNA-Seq data with the Trimmomatic software (v.0.36) (Bolger et al., 2014). We removed adapter sequences and low-quality reads from the raw data. All clean reads were then mapped to the *B. napus* reference genomev.4.1^1^ using the STAR program (v.2.5.3) (Dobin et al., 2013) with default parameters. We then mapped the aligned reads to RNA features using the feature Counts function of Subread (v.1.6.0) (Liao et al., 2014). We identified [differentially expressed gene (DEGs)] with the gene counts generated above with the edgeR package (Robinson et al., 2010) to import, organize, filter, and normalize the data with a false discovery rate (FDR) of 1%. We quantified relative gene expression as fragments per kilobase of exon model per million mapped reads (FPKM) by Cuffdiff within Cufflinks (Trapnell et al., 2012). After calculating their expression fold-change, we identified genes with FDR-adjusted *q*-value ≤ 0.05 and absolute log_2_ (fold-change) ≥ 1 as DEGs.

### Identification of Differentially Expressed Conserved and Novel MicroRNAs

Clean data were obtained from small RNA sequencing libraries by removing adapter sequences from all reads, as well as reads containing over 10% Ns and low-quality (Q20 < 85%) reads from the raw data. We extracted potential small RNAs 18–30 nt in length and mapped them to the *B. napus* reference genome using Bowtie2 (v.2.2.9) (Langmead et al., 2009). Mapped reads were further mapped to the released *B. napus* miRNAs in miRBase22 (v.22.1)^2^ using basic local alignment sequence tool for nucleotides (BLASTN) with an *E*-value cutoff of ≤ 1e-5, which identified known miRNAs. We used the Rfam database (v.14.4)^3^ to remove ribosomal RNAs (rRNAs), transfer RNAs (tRNAs), small nuclear RNAs (snRNAs), and small nucleolar RNAs (snoRNAs). In order to improve the accuracy and efficiency of comparison, we retained only the longest sequence from the remaining reads and merged them with the sequences in miRBase22 to become the final *B. napus* miRNA dataset. After removing the reads already classified as known miRNAs, we employed miRDeep-P (v.1.1.4) (Yang and Li, 2011) to predict potential novel miRNAs, allowing for 2 bp 3′ overhangs. We named these potential novel miRNAs based on the chromosome they map to and their starting nucleotide position on that chromosome.

To identify differentially expressed miRNAs (DEMs), we normalized miRNA expression across all samples using Salmon (v.0.11.3) (Patro et al., 2017) to obtain the expression of transcript per million (TPM) based on the normalization formula: normalized expression = (actual miRNA counts/total number of mapped reads) × 1,000,000. We calculated the associated fold-change, *p*-values, and *q*-values with in-house Perl scripts. miRNAs with FDR-adjusted *q*-value ≤ 0.05 and absolute log_2_ (fold-change) ≥ 1 were determined to be DEMs.

### MicroRNA Target Prediction

We employed the psRNATarget server (v. 2017)^4^ to predict the target genes for all known and novel miRNAs that were differentially expressed between comparable groups, with default parameters.

### Gene Function Clustering Analysis

Functional annotation was performed by using BLASTX to compare *B. napus* and Arabidopsis proteins. We used the online tool agriGO (gene ontology analysis toolkit and database for agricultural community, v.2.0)^5^ (Tian et al., 2017) for GO enrichment analysis. We determined GO classifications by submitting the gene sequences to the BLAST4ID tool of agriGO to obtain the corresponding *B. napus* locus ID, and then running GO term enrichment analysis.

### Weighted Gene Co-expression Network Analysis

Weighted gene co-expression network analysis (WGCNA) package in R was designed in 2008 for helping users create weighted correlation network modules and identify key genes associated with traits in interesting modules (Langfelder and Horvath, 2008). FPKM values of genes were log_2_ (FPKM + 1) transformed for further calculation of correlation coefficient, determination of gene modules, construction of co-expression network, and correlation analysis of modules and phenotypic traits. In the process of analysis, the soft thresholding power was determined using the pickSoftTreshold function based on the scale-free topology model fit (R^2^) > 0.9; the automatic blockwiseModules network construction approach was applied to obtain the highly correlated modules, with the following parameters: power = 7; maxBlockSize = 30,000; TOM-type = unsigned; miniModuleSize = 30; reassignThreshold = 0, mergeCutHeight = 0.25. The co-expression networks were displayed using Cytoscape (v.3.5.1) (Maere et al., 2005).

### Validation of Transcript Levels by Quantitative Reverse Transcription-PCR

The same RNA samples prepared for mRNA and miRNA sequencing libraries were used for cDNA synthesis and quantitative reverse transcription-PCR (qRT-PCR) detection. qRT-PCR was performed as described previously (Qu et al., 2015). We used gene-specific primers obtained from qPrimerDB^6^ (Lu et al., 2018). Relative transcript levels were normalized to the *B. napus* housekeeping genes *UBIQUITIN-CONJUGATING ENZYME 21* (*Bna.UBC21*) and *Bna.ACTIN7* (Qu et al., 2016).

For the validation of miRNAs, we added a poly (A) tail and performed reverse transcription from 2-μg RNA in 20-μl reaction volume using the miRcute miRNA First-Strand cDNA Synthesis Kit (Tiangen, Beijing, China). We then diluted the cDNAs 10-fold for RT-qPCR analysis, using the miRcute miRNA qPCR Detection Kit (SYBR) (Tiangen, Beijing, China). We carried out PCR reaction solutions containing 1-μl (∼10 ng) diluted cDNA, 10-μl 2- × -miRcute miRNA premix, a 0.4-μl forward primer (10 μM), a 0.4-μl reverse primer (10 μM), and 8.2-μl ddH_2_O on a BIO-RAD CFX96 Real-Time System (BIORAD, United States). Cycle conditions were: 95°C for 15 min, followed by five cycles of 94°C for 20 s, 65°C for 30 s, and 72°C for 34 s, and then 40 cycles of 94°C for 20 s and 60°C for 30 s. All reactions were performed in triplicate, with the *B. napus* U6 snRNA as internal control. Relative gene expression was calculated using the 2*^–ΔΔ*Ct*^* method (Livak and Schmittgen, 2001). All above-mentioned qRT-PCR assays were performed according to MIQE guidelines (Bustin et al., 2010). Three independent biological replicates, each with three technical replicates, were implemented for each sample.

## Results

### Physiological Characteristics of Accessions With Different Harvest Index

In the context of this study, we planted the *B. napus* accessions YC24, YC52, and YC46 at the Beibei, Chongqing (CQ), and Lincang, Yunnan (YN) locations during the 2015–2016 growing season. All phenotypic values are provided in Table 1; in order to distinguish experimental groups, they were named as planting location first (CQ or YN) plus the accession number (24, 52, or 46). We collected HI-related phenotypic traits, such as HI, biomass yield per plant (BY), seed yield per plant (SY), stem dry weight (ST), canopy biomass yield (CBY). We noticed that the HI for all accessions significantly increased at the YN location when compared to that measured at CQ, especially accessions YC24 and YC52. In addition, YC24 displayed a significantly higher HI than the YC52 and YC46 accessions at both locations (Table 1); therefore, YC24 was regarded as a high HI accession, and YC52 and YC46 as low HI accessions. We used these three accessions to elucidate the molecular mechanism of HI.

**TABLE 1:** Phenotypic values of the three varieties measured in Chongqing and Yunnan.

	CBY (g)	SY (g)	ST (g)	BY (g)	HI (%)
CQ24	68.8 ± 4.0cd	29.6 ± 3.8cd	50.8 ± 6.1bc	119.6 ± 9.5bc	24.8 ± 2.7c
CQ52	75.9 ± 3.4c	25.5 ± 0.3d	54.2 ± 6.6bc	130.1 ± 4.0b	19.6 ± 0.5d
CQ46	56.0 ± 5.4d	15.0 ± 1.6e	42.6 ± 3.0c	98.6 ± 8.4c	15.2 ± 0.9e
YN24	132.2 ± 12.7a	62.8 ± 6.2a	61.1 ± 14.8b	193.3 ± 27.5a	32.6 ± 1.4a
YN52	120.6 ± 3.5a	52.8 ± 2.4b	66.2 ± 6.4b	186.8 ± 5.5a	28.2 ± 0.8b
YN46	102.8 ± 17.1b	35.3 ± 7.3c	86.8 ± 10.1a	189.7 ± 26.4a	18.5 ± 1.7d

*The data are means ± SD; columns with different letters indicate significant differences based on Duncan’s multiple range tests at p < 0.05.*

### mRNA Sequencing Data Analysis Uncovers Harvest Index-Related Differentially Expressed Genes

We collected 48 samples for RNA extraction and subsequent deep sequencing of the transcriptome (RNA-Seq) analysis: ZS, SP, Le, and St in biological duplicates from all three accessions at the two geographic locations. After removal of low-quality reads from the raw data, we retained 40.29∼52.68 million clean reads; 83.97 to 95.23% of which were mapped to the *B. napus* reference genome (except SP in CQ24 Sample 1, which shows just 61.67%). Sample correlation analysis used FPKM to emphasize the high degree of correlation between biological replicates (Supplementary Table 1). The raw sequencing data were deposited in the BIG Data Center under BioProject accession No. PRJNA602979.

**FIGURE 1:**
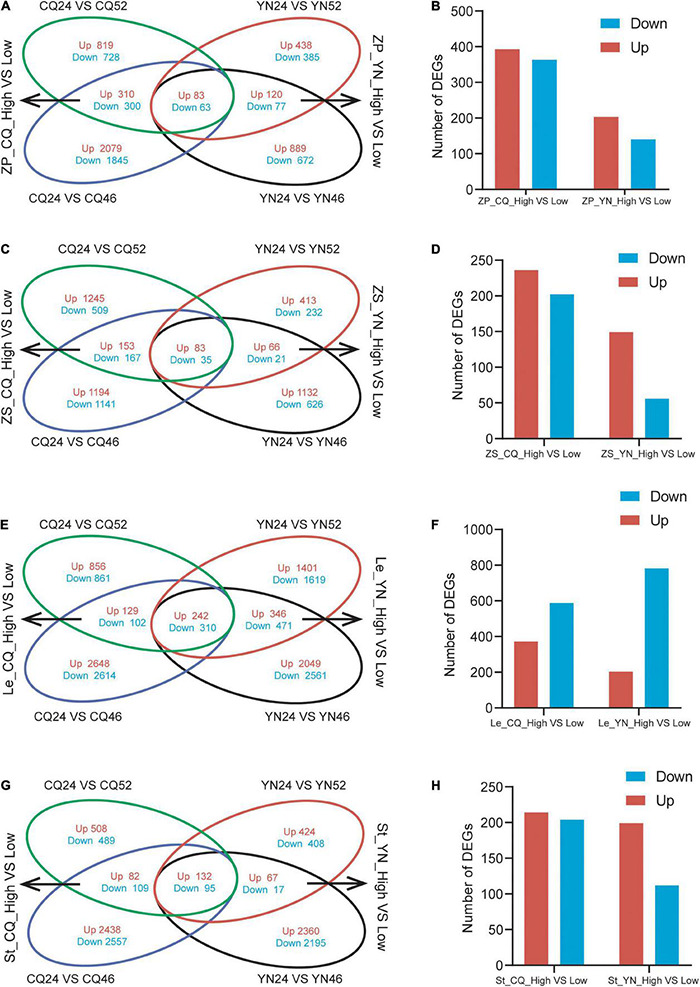
Venn diagrams and the number of DEGs of the DEGs from high- and low-HI accessions. **(A)** A Venn diagram of DEGs between high- and low-HI accessions in ZP at CQ and YN. **(B)** The number of DEGs between high- and low-HI accessions in ZP. **(C)** A Venn diagram of DEGs between high- and low-HI accessions in Le at CQ and YN. **(D)** The number of DEGs between high- and low- HI accessions in Le. **(E)** A Venn diagram of DEGs between high- and low-HI accessions in ZS at CQ and YN. **(F)** The number of DEGs between high- and low-HI accessions in ZS. **(G)** A Venn diagram of DEGs between high- and low-HI accessions in ST at CQ and YN. **(H)** The number of DEGs between high- and low-HI accessions in ZP in ST.

We identified DEGs across SP samples; we detected 2,303 DEGs (1,212 upregulated and 1,091 downregulated) in CQ24 vs. CQ52; 4,680 DEGs (2,472 upregulated and 2,208 downregulated) in CQ24 vs. CQ46; 1,103 DEGs (641 upregulated, 462 downregulated) in YN24 vs. YN52; 1,904 DEGs (1,092 upregulated, 812 downregulated) in YN24 vs. YN46 (Supplementary Table 2). To distinguish HI-related DEGs between high- and low-HI lines, we generated a four-way Venn diagram showing the extent of overlap between the four comparisons listed above. This analysis highlighted 756 DEGs (393 upregulated and 363 downregulated) in SP between high- and low-HI accessions grown at the CQ location, and 343 DEGs (203 upregulated and 140 downregulated) between high- and low-HI accessions grown at the YN location. A subset of 146 DEGs (83 upregulated and 63 downregulated) was identified in SP samples collected at both locations and across all accessions (Figures 1A,B).

We repeated the same analysis with the RNA-Seq data obtained from the ZS samples; we identified 2,192 DEGs (1,481 upregulated, 711 downregulated) in CQ24 vs. CQ52; 2,776 DEGs (1,430 upregulated and 1,346 downregulated) in CQ24 vs. CQ46; 850 DEGs (562 upregulated, 288 downregulated) in YN24 vs. YN52; 1,963 DEGs (1,281 upregulated, 682 downregulated) in YN24 vs. YN46 (Supplementary Table 2). The corresponding four-way Venn diagram showed that 438 DEGs (236 upregulated and 202 downregulated) were shared by high- and low-HI accessions grown at the CQ location, while 205 DEGs (149 upregulated and 56 downregulated) were common to high- and low-HI accessions grown at the YN location. Furthermore, 118 genes (83 upregulated and 35 downregulated) were differentially expressed in seed samples collected at both locations and across all accessions (Figures 1C,D).

Leaves are the same “source” organs as the SP; through RNA-seq data, we identified 2,500 DEGs (1,227 upregulated, 1,273 downregulated) in CQ24 vs. CQ52; 6,047 DEGs (3,019 upregulated and 3,026 downregulated) in CQ24 vs. CQ46; 4,660 DEGs (2,185 upregulated, 2,475 downregulated) in YN24 vs. YN52; 6,250 DEGs (2,833 upregulated, 3,417 downregulated) in YN24 vs. YN46 (Supplementary Table 2). Our 4-way Venn diagram showed that 783 DEGs (371 upregulated and 412 downregulated) were shared by high- and low-HI accessions grown at the CQ location, while 1,369 DEGs (588 upregulated and 781 downregulated) were common to high- and low-HI accessions grown at the YN location. And 552 (242 upregulated and 310 downregulated) genes were differentially expressed in Le samples collected at both locations and across all accessions (Figures 1E,F).

Stem as the “flow” organ, which is the limitation for the accumulation of assimilates in *B. napus* seeds (Shen et al., 2010; Fu and Zhou, 2013; Luo et al., 2015), we also analysis stem RNA-seq data; there are 1,415 DEGs (722 upregulated, 693 downregulated) in CQ24 vs. CQ52; 5,313 DEGs (2,652 upregulated and 2,761 downregulated) in CQ24 vs. CQ46; 1,143 DEGs (623 upregulated, 520 downregulated) in YN24 vs. YN52; 4,866 DEGs (2,559 upregulated, 2,307 downregulated) in YN24 vs. YN46 (Supplementary Table 2). And 418 DEGs (214 upregulated, 204 downregulated) in St between high- and low-HI accessions grown at the CQ location; 311 DEGs (199 upregulated, 112 downregulated) in St between high- and low-HI accessions grown at the YN location. About 227 (132 upregulated, 95 downregulated) genes were differentially expressed in St samples collected from the two locations (Figures 1G,H).

Overall, the transcriptome of the accessions YC24 and YC46 differed by more DEGs than when YC24 was compared to the YC52 accession, both in SP, Le, ZS, and St. In addition, SP and Le samples as “source” organs were characterized by more DEGs than St and ZS samples between high- and low-HI accessions at both locations, indicating that the regulation of HI in “source” organs might be more complicated. Finally, samples harvested at the CQ location exhibited more DEGs than at the YN location in SP, ZS, and St, except Le, suggesting that the seed-filling process might be more complex at the CQ location.

In addition, in order to compare the gene expression differences of the same material in different regions, we compared the DEGs of the same material grown in YN and CQ. The results indicate that these DEGs among the three materials in four different plant tissues are quite different in different environments and the expression levels also varied greatly (Supplementary Table 2), especially in the Le of material YC24; there were 4,599 upregulated DEGs and 4,659 downregulated DEGs (Supplementary Figure 2). At the same time, the DEGs in the same tissue of the three materials also have differences; only 16, 36, and 34 same DEGs were found in the St, Le, and SP, and the absence of the same DEGs was found in the ZS (Supplementary Figure 2). Moreover, combining with Table 1, we found that HI in YN was significantly higher than that in CQ, indicating that HI was easily affected by environmental conditions; this is worthy of further study.

### Functional Annotation and Classification of Differentially Expressed Genes

To understand the functions encoded by the DEGs identified between high- and low-HI accessions, we performed a GO enrichment. Studies showed long ago that the photosynthates of the SP are the main sources of seed yield, contributing about 2/3 of the total dry matter of the seed yield, whereas the beak of the seeds is about 8% (Leng et al., 1992), which can seriously affect HI, so we focused on SP and ZS first.

Differentially expressed genes in SP, harvested at the CQ location between high- and low-HI accessions (SP_CQ_High vs. Low), were associated with 93 significantly enriched GO terms (*q*-value ≤ 0.05), while DEGs for the equivalent samples collected at the YN location (SP_YN_High vs. Low) showed a significant enrichment for 341 GO terms; in addition, we identified 54 significantly enriched GO terms (*q*-value ≤ 0.05) for SP among DEGs shared by both CQ and YN locations (Supplementary Figures 3A,B). We observed a significant enrichment in several cellular component GO terms related to “chloroplasts” (GO: 0005737, GO: 0009507, GO: 0009536, GO: 0044434, GO: 0044435), and we noticed that GO terms of SP in addition to response to biological stress and a large number of abiotic stresses (GO:0009628, response to abiotic stimulus; GO:0009408, response to heat; GO:0009607, response to biotic stimulus; etc.); there are some interesting pathways enriched in “circadian rhythm” (GO:0007623), “histone H3-K36 demethylation” (GO:0070544), and “response to Karrikin” (GO:0080167), which suggest that circadian rhythm, histone modification affected the plant HI; also, as Karrikin, a new signaling molecule participates in regulating HI (Supplementary Figure 3C).

**FIGURE 2:**
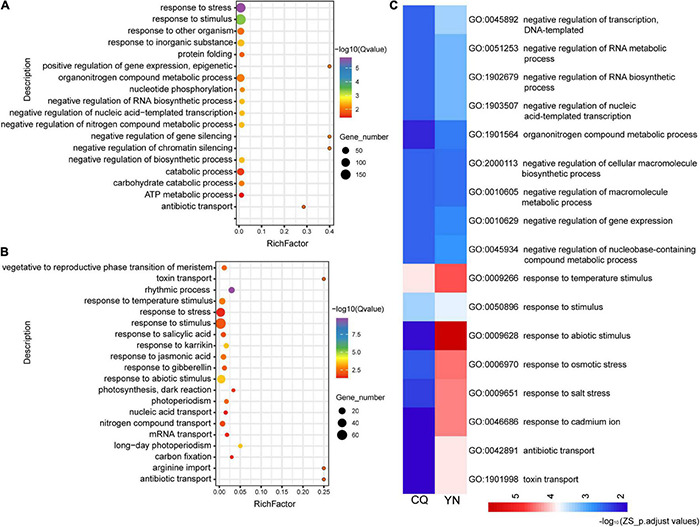
GO functional classification of the ZS DEGs from high- and low-HI accessions at CQ and YN. **(A)** ZS_CQ_High vs. Low. **(B)** ZS_YN_High vs. Low. **(C)** Common GO terms shared by CQ and YN varieties. The color bars under the figures represent -log_10_ (*p*.adjust values).

In ZS samples, a similar analysis revealed 107 significantly enriched GO terms among DEGs between high- and low-HI accessions harvested at the CQ location (ZS_CQ_High vs. Low), and 59 significantly enriched GO terms among DEGs between high- and low-HI accessions harvested at the YN location (ZS_YN_High vs. Low) (Figures 2A,B). We identified 18 common enriched GO terms among DEGs for ZS. Biological processes-related GO terms, such as the “negative regulation of RNA metabolic process” (GO: 1902679), “negative regulation of nucleic acid-templated transcription” (GO: 1903507), “negative regulation of the RNA metabolic process” (GO: 0051253), and the “organonitrogen compound metabolic process” (GO: 1901564), were predominantly enriched in ZS samples harvested at both CQ and YN locations (Figure 2C). In the YN location, we noticed “circadian rhythm” (GO: 0007623) and “alternative mRNA splicing *via* spliceosome” (GO: 0000380) were identified but not identified in the CQ location. We suspected that, in ZS samples, circadian rhythm and alternative mRNA splicing may participate in the HI regulation but were also influenced by geographical differences and environmental factors.

GO enrichment analysis of St and Le DEGs in the CQ location obviously enriched in “circadian rhythm” (GO: 0007623) as the results in SP and ZS; we speculated that circadian rhythm plays an important role in regulating HI. We also found St and Le DEGs are mainly enriched in hormone-related terms, such as the “ethylene-mediated signaling pathway” (GO: 0009873), “jasmonic acid-mediated signaling pathway” (GO: 0009867) in St at the CQ location, “response to gibberellin stimulus” (GO: 0009739) in St at the YN location. In Le samples, “response to gibberellin stimulus” (GO: 0009739) and the auxin metabolic/biosynthetic process (GO: 0009850 and GO: 0009851) also enriched, suggesting that, although the leaf no longer provides most of the photosynthetic energy during silique ripening (SP provides more), it works with the St to regulate plant growth hormonally (Supplementary Figure 4).

### Construction of Co-expression Networks

**FIGURE 3:**
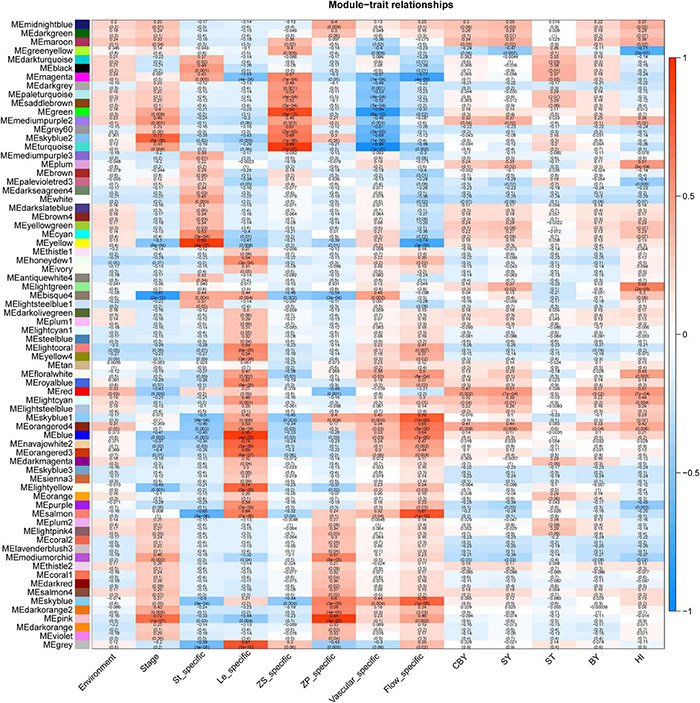
An overview of module and traits corresponding through WGCNA.

Through WGCNA, we constructed co-expression networks with all DEGs and 13 phenotypic data (Supplementary Table 3). This analysis yielded 72 gene modules, each represented by different colors in the output (Figure 3). The smallest module is the ME light coral, which included only 55 genes, and the largest module is turquoise, which included 10,008 genes. We focused on the ME light green module that is highly correlated with the HI (*r*^2^ = 0.61).

GO enrichment analysis showed the ME light green module was mainly enriched in the “ubiquitin-dependent protein catabolic process” (GO: 0006511), “entrainment of the circadian clock” (GO: 0009649), “positive regulation of meiosis” (GO: 0045836), and “production of miRNAs involved in gene silencing by miRNA” (GO: 0035196). This indicated that ubiquitin modification, circadian rhythm, and gene silencing are all strong connection with HI; the results are the same with our previous DEGs analyzed. We used the maximal clique centrality method in the cytoHubba plugin of Cytoscape (v. 3.5.1) to identify hub genes in the ME light green module of interest, and we identified *BnaA07g02330D* (*Bna.A07STY46*), which is serine/threonine kinase that phosphorylates transit peptides of chloroplast and mitochondria-targeted pre-proteins and is involved in chloroplast differentiation in Arabidopsis (Giorgia et al., 2011). We speculated that *Bna.A07STY46* may participate in retarded establishment of a photosynthetic capacity to influence HI.

### Identification of Differentially Expressed MicroRNAs Based on MicroRNA Sequencing

In addition to 48 mRNA libraries, we also sequenced 12 miRNA libraries of samples SP and ZS. We obtained 163,448,662 reads and retained 159,966,665 clean reads after removing low-quality reads and adapters (Supplementary Table 4). We then selected clean reads with a length of 18–30 nt for further analysis. As expected, reads with a length of 21, 22, and 24 nt were more abundant out of all reads. In addition, we observed a higher fraction of 21-nt reads in silique pericarp samples when compared to that in seed samples, while 24-nt reads showed the opposite pattern (Supplementary Figure 5). We identified 903 miRNAs from the 12 miRNA libraries, including 46 known miRNAs and 857 novel miRNAs (Supplementary Table 5).

Based on the selection criteria of an absolute log_2_ FC > 1 and a *q*-value < 0.05, we detected DEMs between the same comparison groups as for RNA-Seq analysis. When we compared SP_CQ_High and Low, we identified 11 known DEMs (1 upregulated and 10 downregulated) and 86 novel DEMs (60 upregulated and 26 downregulated), while the comparison of SP_YN_High with Low revealed 15 known DEMs (0 upregulated and 15 downregulated) and 80 novel DEMs (38 upregulated and 42 downregulated). A comparison between ZS_CQ_High and Low yielded 10 known DEMs (8 upregulated and 2 downregulated) and 70 novel DEMs (38 upregulated and 32 downregulated), while we identified 11 known DEMs (11 upregulated and 0 downregulated) and 65 novel DEMs (30 upregulated and 35 downregulated) from a comparison between ZS_YN_High and Low. In addition, SP samples harvested at the CQ and YN locations shared 8 known DEMs and 21 novel DEMs, and ZS samples collected at the two locations saw an overlap consisting of 8 known DEMs and 16 novel DEMs (Supplementary Figure 6).

### Integration of Differentially Expressed Genes and Differentially Expressed MicroRNAs

To elucidate the regulatory role of DEMs between high- and low-HI accessions, we first identified the potential target genes of each DEM before combining the expression profiles of DEMs and their target genes for further analysis. We thus obtained miRNA–mRNA interaction pairs (pairs with either negatively or positively correlated expression patterns) through a comparison of high- and low-HI accessions; 130 pairs in SP were harvested at CQ, 68 pairs in SP were collected at YN, 69 pairs in ZS from the CQ location, and 23 pairs in ZS from the YN location. Overall, almost half of the miRNA–mRNA interactions pairs showed a negative correlation, as might be expected from a true mRNA–miRNA pair involving transcript cleavage. We also noticed that several miRNAs had multiple potential target mRNAs, and multiple miRNAs that targeted a single mRNA (Supplementary Figure 7 and Supplementary Table 6). For instance, the upregulated miRNA bna-miRC03_52 controlled the downregulated genes *BnaC07g39100D*, *BnaC08g32460D*, and *BnaA06g21420D*, while the upregulated miRNA bna-miRA02_2282 controlled the downregulated genes *BnaA06g21420D* and *BnaA06g39650D* in comparisons between SP_CQ_High and Low (Supplementary Figure 7). These observations indicate that the regulation of miRNA–mRNA pairs is complex.

**FIGURE 4:**
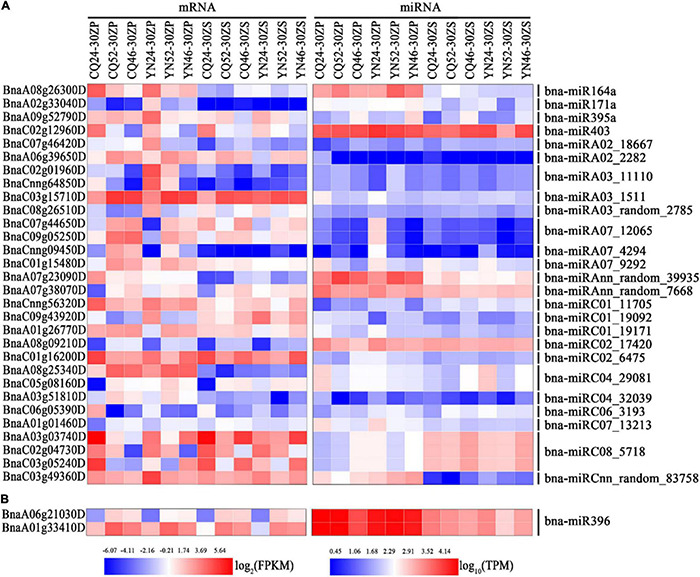
Heatmap representation of interesting miRNA-mRNA pairs identified in silique pericarps and seeds. **(A)** Heatmap representation of miRNA-mRNA pairs-related circadian rhythm, response to stress, response to abiotic stimulus, and response to stimulus in silique pericarps. **(B)** Heatmap representation of *bna*-miR396-modulated miRNA-mRNA pairs in seeds. The color bars under the figures represent log_2_ (FPKM/TPM).

We performed a GO functional annotation analysis to characterize these differentially expressed target genes. Our results showed enrichment in GO terms related to circadian rhythms, transporter activity response to stress, response to abiotic stimulus, and response to stimulus across all comparisons, with the exception of ZS_CQ_High vs. Low (Supplementary Figure 8 and Supplementary Table 7). We hand-selected miRNA–mRNA pairs associated with the GO terms mentioned above, such as the bna-miRC08_5718–*BnaA03g03740D/BnaC03g05240D* pair and the bna-miRC01_19092*–BnaC09g43920D* pair (Figure 4A and Supplementary Figure 8). We did not discover significantly enriched GO terms among miRNA–mRNA pairs from seed samples collected at the CQ and YN locations between high- and low-HI accessions. However, the bna-miR396*–BnaA06g21030D* and bna-miR396*–BnaA01g33410D* pairs were shared between the two locations (Figure 4B and Supplementary Figure 8).

### Validation of mRNA and MicroRNA Expression by Quantitative Reverse Transcription-PCR

**FIGURE 5:**
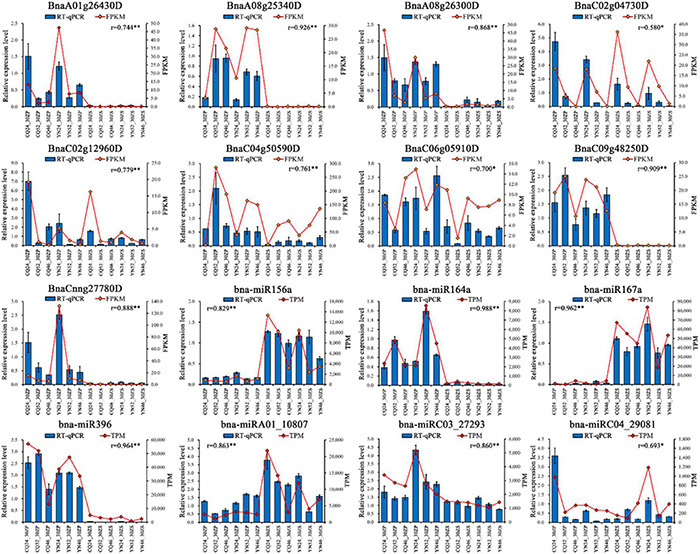
RT-qPCR validation of DEGs and DEMs. Blue bars represent the relative expression level of RT-qPCR, and red points represent FPKM/TPM of sequencing; *r* represents Pearson correlation coefficient, *: correlation is significant at the 0.05 level, **: correlation is significant at the 0.01 level.

To validate the quantification of the mRNA and miRNA sequencing data presented here, we analyzed the relative transcript levels of nine randomly selected DEGs (BnaA01g26430D, *BnaA08g25340D*, *BnaA08g26300D*, *BnaC02g04730D*, *BnaC02g12960D*, *BnaC04g50590D*, *BnaC06g05910D*, *BnaC09g48250D*, and *BnaCnng27780D*) and 7 DEMs (bna-miR156a, bna-miR164a, bna-miR167a, bna-miR396, bna-miRA01_10807, bna-miRC03_27293, and bna-miRC04_29081) by qRT-PCR on the same RNA used for library construction (Supplementary Table 8 and Figure 5). We observed a high degree of positive correlation between the relative transcript levels of DEGs and DEMs determined by qRT-PCR and their relative expression measured from high-throughput sequencing data. The high-throughput sequencing data used in this study were, therefore, accurate and reliable.

## Discussion

Harvest index is a complex agronomic trait of great economic value that depends on interactions between a plant genotype and the environment (Amanullah and Shah, 2010; Unkovich et al., 2010; Gajića et al., 2018; Chao et al., 2019). We determined that the YC24 accession showed a significantly higher HI than the YC52 accession, while the YC52 accession had a significantly higher HI than the YC46 accession at both the CQ and YN locations. These results indicated that HI differences between the YC24, YC52, and YC46 accessions were robust, validating the use of these three accessions to dissect the regulatory mechanism behind HI. YN is a high-yield crop production environment; not surprisingly, HI for the YC24, YC52, and YC46 accessions grown at this location was higher than that from the CQ location, indicating that environmental conditions can have a strong influence on HI. Thus, we carried out transcriptome sequencing to compare the gene expression profile in accessions grown in a standard HI (CQ) and high-HI (YN) environment during the seed-filling stage.

### RNA-Seq and Expression Profiles of High- and Low-Harvest Index Materials

Based on gene functional annotation data, we identified significantly enriched GO terms associated with DEGs between high- and low-HI accessions. In *B. napus*, SP are photosynthetically active, and, as source tissues, they thus provide nutrients to seeds, a sink organ (Diepenbrock, 2000; Bennett et al., 2011). Furthermore, SP mediate maternal control during seed filling (Li et al., 2019). We also noticed that a large fraction of enriched GO terms were specific to the CQ location, as we did not detect them in our analysis of silique pericarp or seed samples collected at the YN location, underscoring the marked environmental sensitivity of HI regulation.

As HI represents the ratio between reproductive organs and vegetative biomass produced, regulating the timing of the phase transition from vegetative to reproductive growth will be beneficial to increasing HI (Hütsch and Schubert, 2017). Based on GO enrichment analysis, we noticed that the circadian rhythm may be associated with HI. In Arabidopsis, the core circadian rhythm comprises the proteins CIRCADIAN CLOCK ASSOCIATED 1 (CCA1), LATE ELONGATED HYPOCOTYL (LHY), TIMING OF CAB EXPRESSION 1 (TOC1), and PSEUDO RESPONSE REGULATORS (PRRs) (Mcclung and Gutiérrez, 2010). In addition, the *CONSTANS* gene family (*CO*) controls photoperiodic flowering time together with *PRRs* (Nakamichi et al., 2007). Transcriptome analysis revealed that genes associated with circadian rhythm were potentially involved in potato (*Solanum tuberosum*) tuber formation, suggesting that the circadian rhythm may participate in photoassimilate distribution (Shan et al., 2013). In rice, *CONSTANS-like* (*OsCOL9*) not only modulated photoperiodic flowering but influenced grain number of the main panicle (Liu et al., 2016). Similarly, *TOC1* modulated chickpea (*Cicer arietinum*) seed yield per plant (Basu et al., 2019), while overexpression of Arabidopsis *PRR5* in rice delayed flowering but also significantly increased biomass (Nakamichi et al., 2020), likely due to higher expression of *OsPRR37*; the overexpression line GCA1^OX^-5 showed better general combining ability of rice (Liu et al., 2015). In wheat, the *Photoperiod-1* (*Ppd-1*) mutant inactivates a *PRR* and affects paired spikelet formation (Boden et al., 2015). Moreover, our analysis highlighted additional circadian rhythm-related genes [*GLYCINE RICH PROTEIN7* (*GRP7*), *EARLY FLOWERING4* (*ELF4*), *REVEILLE 1* (*RVE1*)] as being differentially expressed between high- and low-HI lines, strongly suggesting that the circadian rhythm and rhythm-controlled gene regulation might be harnessed to modulate HI in *B. napus* in the future.

Under the high-HI environment of the YN location, we detected a number of DEGs expressed in SP that were related to transporter activity, such as *PLASMA MEMBRANE INTRINSIC PROTEIN* (*PIP*), *BILE ACID TRANSPORTER 5* (*BAT5*), *ABC2 HOMOLOG 13* (*ATH13*), *CHLORIDE CHANNEL A* (*CLC-A*), *MAJOR FACILITATOR SUPERFAMILY PROTEIN*, *NITRATE TRANSPORTER 1.7* (*NRT1.7*), *POLYOL/MONOSACCHARIDE TRANSPORTER 5* (*PMT5*), and *VACUOLAR GLUCOSE TRANSPORTER 1* (*VGT1*). PIP are aquaporins that localize to the plasma membrane and facilitate the flux of water and solutes across the plasma membrane (Kourghi et al., 2017; Wang H. et al., 2020). Multiple studies have shown that *PIP* genes can affect water balance and solute transport in plant cells (Sommer et al., 2008; Byrt et al., 2017; Macho-Rivero et al., 2018). The BAT5 transporter translocates glucosinolates, which are derived from methionine and sugars, across the chloroplast membranes (Sawada et al., 2009). ATH13 affects the lipid composition of chloroplast membranes and regulates iron distribution within chloroplasts (Manara et al., 2014, 2015). CLC-A regulates the accumulation of nitrate within vacuoles and plays an important role in modifying cytosolic conditions (De Angeli et al., 2006; Manara et al., 2015; Demes et al., 2020). Additional regulators of nitrate balance and transport include members of the NITRATE TRANSPORTER1/PEPTIDE TRANSPORTER (NPF) family, such as AtNPF3.1 (At1g68570) (Yang et al., 2017) and AtNPF6.2 (At2g26690) (Tong et al., 2016); AtNRT1.7/NPF2.13 (At1g69870) also plays important roles in source-to-sink remobilization of nitrate in Arabidopsis (Fan et al., 2009; Liu et al., 2017). PMT5 can transport a wide range of linear polyols (three to six carbon backbones), cyclic polyols (myo-inositol), pyranose, furanose, hexoses, and pentoses across the plasma membrane (Klepek et al., 2005, 2010). Collectively, the upregulation of genes-encoding proteins with transporter activity in SP of high-HI accessions points to their possible involvement in facilitating water and solute fluxes (e.g., sugar, nitrate nutrients, and cations) from the mother plant to the developing seed, and thus in increasing HI. The only downregulated transporter identified in this study was *VGT1*. However, VGT1 localizes to the vacuolar membrane and mediates the transport of glucose from the cytoplasm to the vacuole (Aluri and Büttner, 2007; Büttner, 2007); a reduction in *VGT1* expression may, therefore, promote sugar translocation to sink tissues by a modulating glucose flux to the vacuole.

In seeds, GO terms, such as GO: 0045892, GO: 1902679, GO: 1903507, GO: 0051253, GO: 0045934, GO: 0010629, GO: 0010605, and GO: 1901564, were significantly enriched in samples harvested at both the CQ and YN locations. For the latter GO term (the organonitrogen compound metabolic process, GO: 1901564), the *B. napus* gene *Bna*C08g45660D homologous to *At*1g01090 (*PYRUVATE DEHYDROGENASE E1 ALPHA, PDH-E1 ALPHA*) was upregulated in high-HI accessions. As *PDH-E1 ALPHA* has been shown to regulate acyl lipid metabolism (LeClere et al., 2004; Mentzen et al., 2008), we speculated that the upregulation of *Bna*C08g45660D contributes to the accumulation of storage lipids in seeds. Gene functional annotation of the GO terms GO: 0045892, GO: 1902679, GO: 1903507, GO: 0051253, GO: 0045934, GO: 0010629, and GO: 0010605 determined that the expression pattern of *Bna*A05g01050D (homologous to *At*2g46830, *CCA1*) showed the same trend at both the CQ and YN locations. Studies have shown that *CCA1* affects not only the circadian rhythm but also the accumulation of storage lipids (Kim et al., 2019). Thus, we concluded that genes that promote the accumulation of storage materials such as lipids during the seed-filling stage likely contribute to the improvement of HI.

### MicroRNA-Mediated Regulatory Networks Related to Harvest Index

MicroRNAs play versatile roles in plant growth and development *via* miRNA–mRNA interaction networks. In our study, we identified abundant miRNA–mRNA interaction pairs from the comparison of silique pericarp and seed samples from high- and low-HI accessions at CQ and YN locations. Based on the functional analysis of target DEGs, we noticed enrichments for genes in silique pericarp samples harvested at both CQ and YN locations related to responses to stimulus. Thus, we selected genes related to circadian rhythm, response to stress, response to abiotic stimulus, and response to stimulus that also exhibited anti-correlations with their miRNAs for further analysis. MiR164 has been shown to control axillary meristem and floral organogenesis formation in Arabidopsis (Raman et al., 2008; Huang et al., 2012); in our study, we observed a negative correlation between bna-miR164a and *BnaA08g26300D*. *BnaA08g26300D* shows homology to the Arabidopsis gene *At1g09560* (*GERMIN-LIKE PROTEIN 5*, *GLP5*). GLP may control resource allocation between primary and lateral roots by phloem-mediated transport in Arabidopsis (Ham et al., 2012), suggesting that the regulation of *GLP5* transcript levels by bna-miR164a may adjust the balance of resources between SP and seeds though the phloem. At5g06530 (ABC TRANSPORTER GENE 22, AtABCG22) also contributes to water transpiration and drought tolerance in Arabidopsis (Kuromori et al., 2011). Hence, we propose that its *B. napus* homolog *BnaC02g01960D*, whose transcript levels are regulated by bna-miRA03_11110, might be important for the proper water balance of SP during the seed-filling phase.

The circadian rhythm gene *TOC1* contributes to energy metabolism by influencing the phase of the circadian rhythm under environmental fluctuations (Legnaioli et al., 2009; Fung-Uceda et al., 2018) and Arabidopsis; *toc1* mutants showed a modified pattern of starch mobilization under light–dark cycles (Flis et al., 2019). We hypothesized that the bna-miRA07_12065*–BnaC09g05250D* (*TOC1*) pair might similarly affect the phase of the circadian rhythm to modulate resources allocation in SP. *BnaC09g43920D* is homologous to *At5g13170* (*SENESCENCE-ASSOCIATED GENE 29*, *SAG29*, also named *SWEET15*). In this study, this gene is upregulated in high-HI SP at the YN location *via* the downregulation of bna-miRC01_19092. SWEET15, together with the transporters SWEET11 and SWEET12, mediates sucrose efflux both intracellularly and intercellularly during seed filling in Arabidopsis (Chen et al., 2015; Eom et al., 2015) and may also regulate senescence under environmental stress (Seo et al., 2011). Moreover, we noted the upregulation of several heat stress-related genes, such as *HEAT SHOCK PROTEIN* (*HSP*) *Hsp17.6CII* (*Bna*A03g03740D/*Bna*C03g05240D), *HSP26.5* (*Bna*C06g05390D), *CPHSC70-1* (*Bna*C01g16200D), and *HEAT SHOCK FACTOR 4* (*HSF4*) (*Bna*Cnng56320D), this upregulation being mediated by the downregulated miRNAs bna-miRC08_5718, bna-miRC06_3193, bna-miRC02_6475, and bna-miRC01_11705 in high-HI lines, respectively. Overall, we identified many miRNA–mRNA pairs related to environmental stress in the current study, indicating that increasing plant adaptability to the environment may adjust resources distribution and improve HI.

Similar to our observation with DEGs, numerous miRNA–mRNA pairs identified here differed between seeds and SP, indicating distinct regulatory mechanisms during the seed-filling stage. Among miRNA–mRNA pairs, *bna-*miR396 and its putative targets *BnaA06g21030D* and *BnaA01g33410D* were shared by seed samples harvested from both the CQ and YN locations. MiR396 exerts a strong influence in plant development by regulating complex traits; for example, miR396 regulates the transcript levels of the *GRF* group to modulate cell proliferation and elongation (Ercoli et al., 2016), grain size and yield (Che et al., 2015; Li et al., 2016; Chen X. L. et al., 2019), and somatic embryogenesis (Szczygieł-Sommer and Gaj, 2019). Moreover, a loss of function in miR396ef resulted in higher grain size and altered plant architecture in rice (Miao et al., 2020), while over-expression of *oa-*miR396c in rice reduced salt and alkali stress tolerance (Gao et al., 2010). In tomato (*Solanum lycopersicum*), over-expressing a short tandem repeat mimic for miR396a (*STTM396a/396a-88*) resulted in earlier flowering and bigger fruits (Cao et al., 2016). In Arabidopsis, the peanut witches’ broom effector PHYLLODY SYMPTOMS 1 (PHYL1) interferes with miR396-mediated regulation of *SHORT VEGETATIVE PHASE* (*SVP*) transcript levels to control flower formation (Yang et al., 2015).

In this study, we also identified the bna-miR396–*BnaA06g21030D*/*BnaA01g33410D* pair. BnaA06g21030D (SER/ARG-rich protein 34A, SRp34a) encodes a member of the Ser-Arg-rich (SR) protein family, which plays multiple roles in post-transcriptional regulation of gene expression by alternative splicing (Sanford et al., 2004; Richardson et al., 2011). BnaA01g33410D is homologous to EMBRYO DEFECTIVE 3119 (EMB3119), which plays an important role in Arabidopsis growth and development (Meinke, 2020). Although many studies have focused on miR396, the roles of the bna-miR396–*Bna.A06SRp34a*/*Bna.A01EMB3119* pair are largely unknown in the context of HI and should be studied in more detail.

**FIGURE 6:**
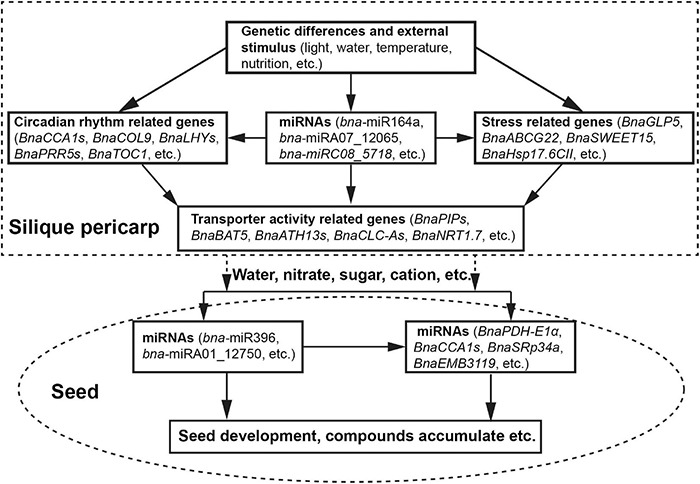
A proposed model for HI determination during the seed-filling stage.

Based on our collective results, we propose a potential model for the regulation of HI in *B. napus* during the seed-filling stage (Figure 6). Genetic differences and external environmental stimuli affect the expression of circadian rhythm-related genes, stress response genes, and miRNAs. miRNAs, in turn, may modulate circadian rhythm-related genes and stress response genes *via* a transcript cleavage. The resulting modulation of transcript levels of transporter activity-related genes will affect water and solutes flow to the seeds, driving seed development and the accumulation of storage compounds, thus determining seed yield and HI.

## Data Availability Statement

The datasets presented in this study can be found in online repositories. The names of the repository/repositories and accession number(s) can be found below: BIG Data Center under BioProject accession number PRJNA602979.

## Author Contributions

JL and KL designed the experiments. CZ, WC, XL, BY, LZ, and ZX performed the bioinformatics analysis. BY and LZ performed RNA extractions and qRT-PCR experiments. CZ and WC wrote the manuscript. All the authors reviewed the manuscript.

## Conflict of Interest

The authors declare that the research was conducted in the absence of any commercial or financial relationships that could be construed as a potential conflict of interest.

## Publisher’s Note

All claims expressed in this article are solely those of the authors and do not necessarily represent those of their affiliated organizations, or those of the publisher, the editors and the reviewers. Any product that may be evaluated in this article, or claim that may be made by its manufacturer, is not guaranteed or endorsed by the publisher.

## Footnotes

1
http://www.genoscope.cns.fr/brassicanapus/data/

2
http://www.mirbase.org/

3
http://rfam.xfam.org/

4
http://plantgrn.noble.org/psRNATarget/

5
http://systemsbiology.cau.edu.cn/agriGOv2/

6
https://biodb.swu.edu.cn/qprimerdb/
