# Supplementary material for: Transcriptome and Small RNA Sequencing Reveal the Mechanisms Regulating Harvest Index in Brassica napus
Source: Front Plant Sci. 2022 Apr 4;13:855486. doi: 10.3389/fpls.2022.855486 (PMC9014204; doi:10.3389/fpls.2022.855486)
Supplement: Supplementary file 9 [file Data_Sheet_1.DOCX]

Supplementary Material

## 1. Supplementary Figures


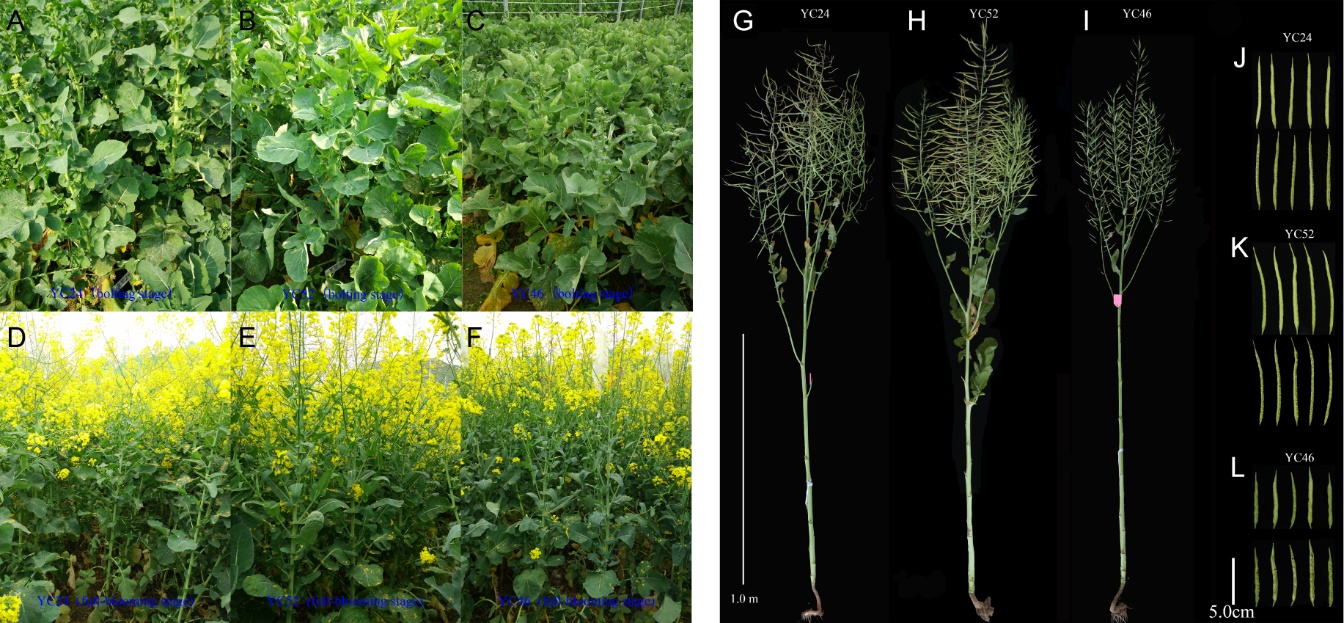


**Supplementary Figure 1.** Phenotypic analysis of the materials YC24, YC52 and YC46 in *Brassica napus.* (A) YC24 (bolting stage). (B) YC52 (bolting stage). (C) YC46 (bolting stage). (D) YC24 (full-blooming stage). (E) YC52 (full-blooming stage). (F) YC46 (full-blooming stage). (G) YC24 (maturity stage). (H) YC52 (maturity stage). (I) YC46 (maturity stage). (J) YC24 (silique). (K) YC52 (silique). (L) YC46 (silique).


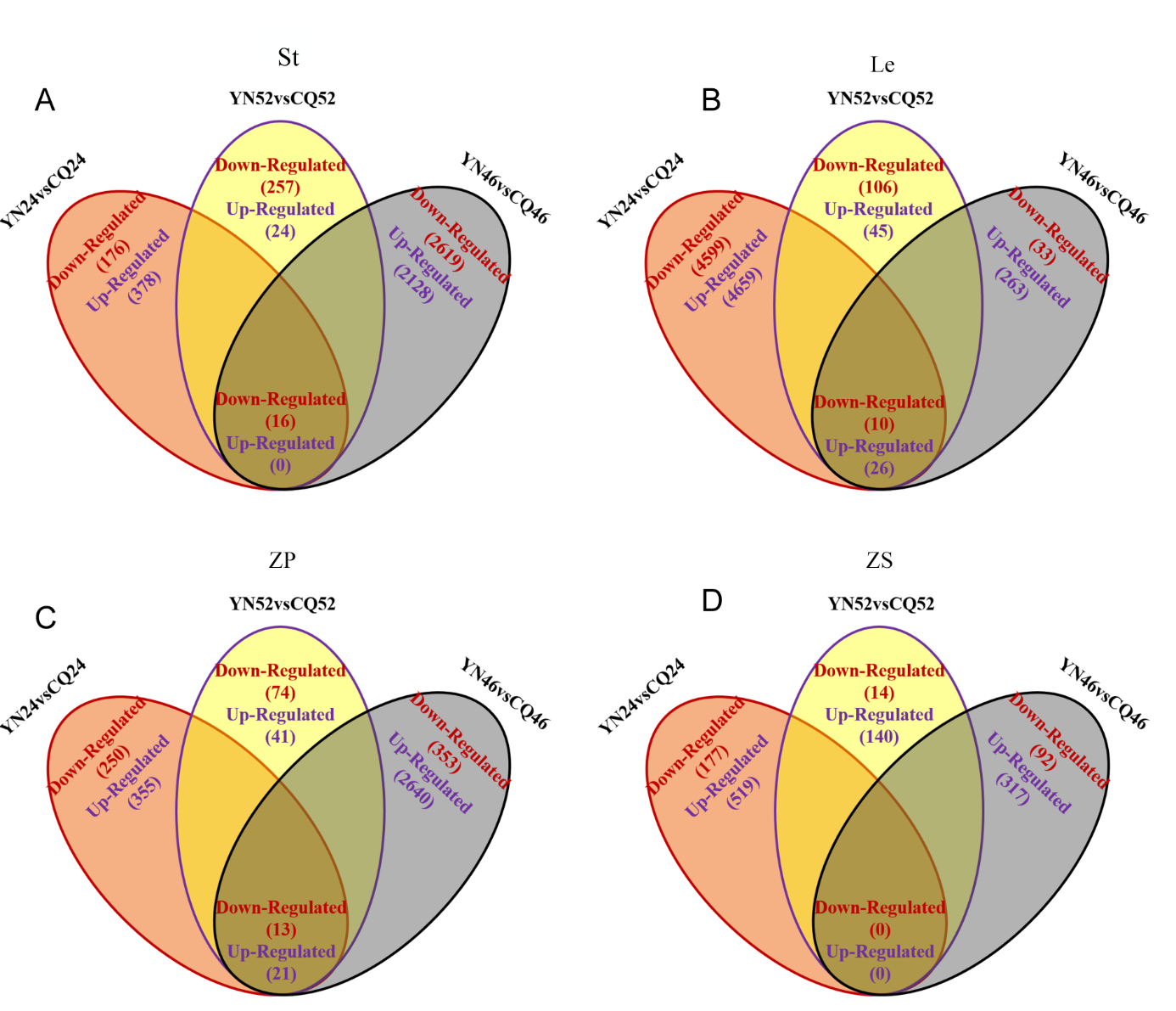


**Supplementary Figure 2.** Venn diagram analysis of DEGs in three materials under different environment. (A) St. (B) Le. (C) ZP. (D) ZS.


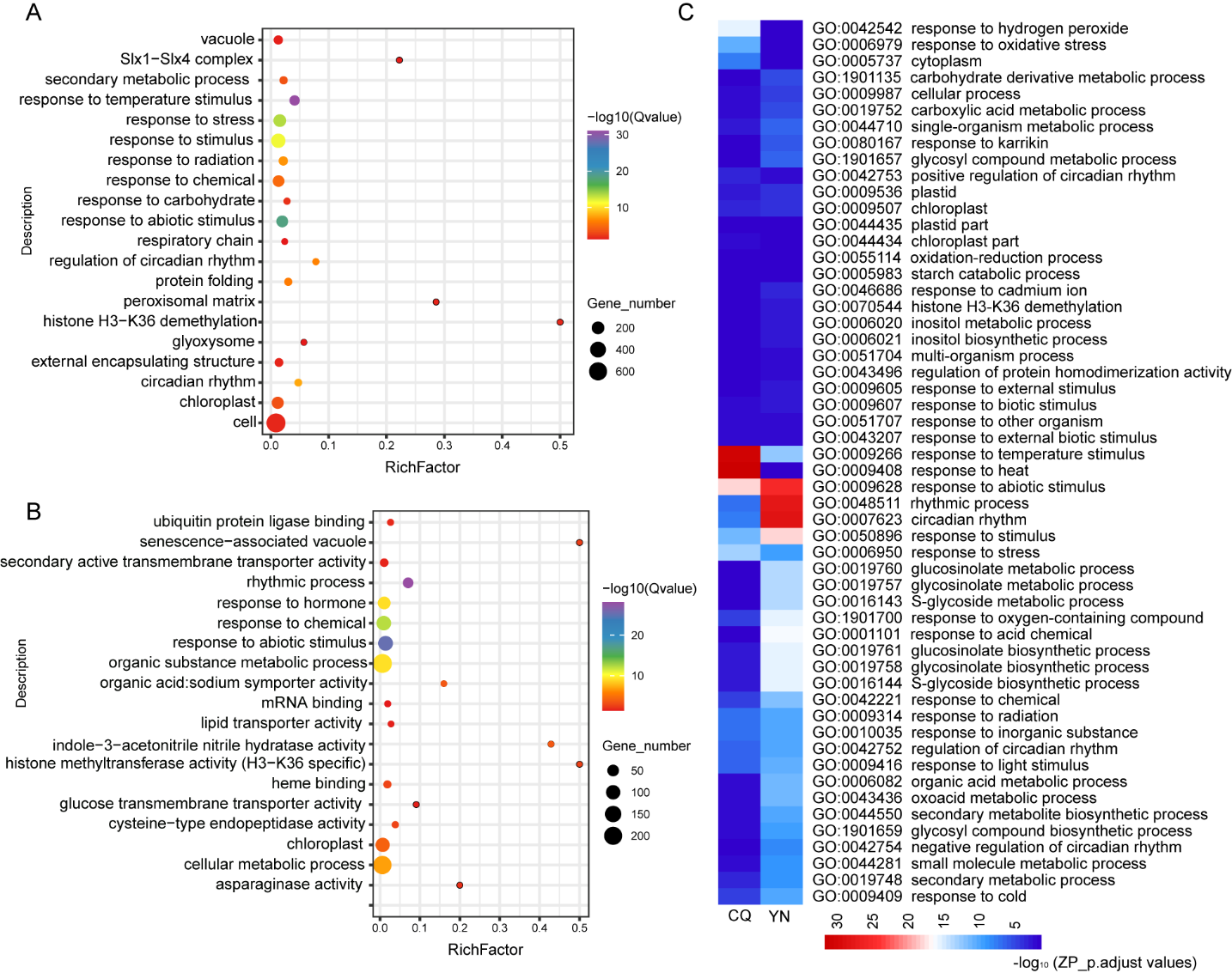


**Supplementary Figure 3.** GO functional classification and common GO terms heatmaps of the ZP DEGs from high- and low- HI accessions at CQ and YN. (A) ZP_CQ_High vs Low. (B) ZP_YN_High vs Low. (C) Common GO terms shared by CQ and YN varieties. The color bars under the figures represent -log_10_ (*p*.adjust values).


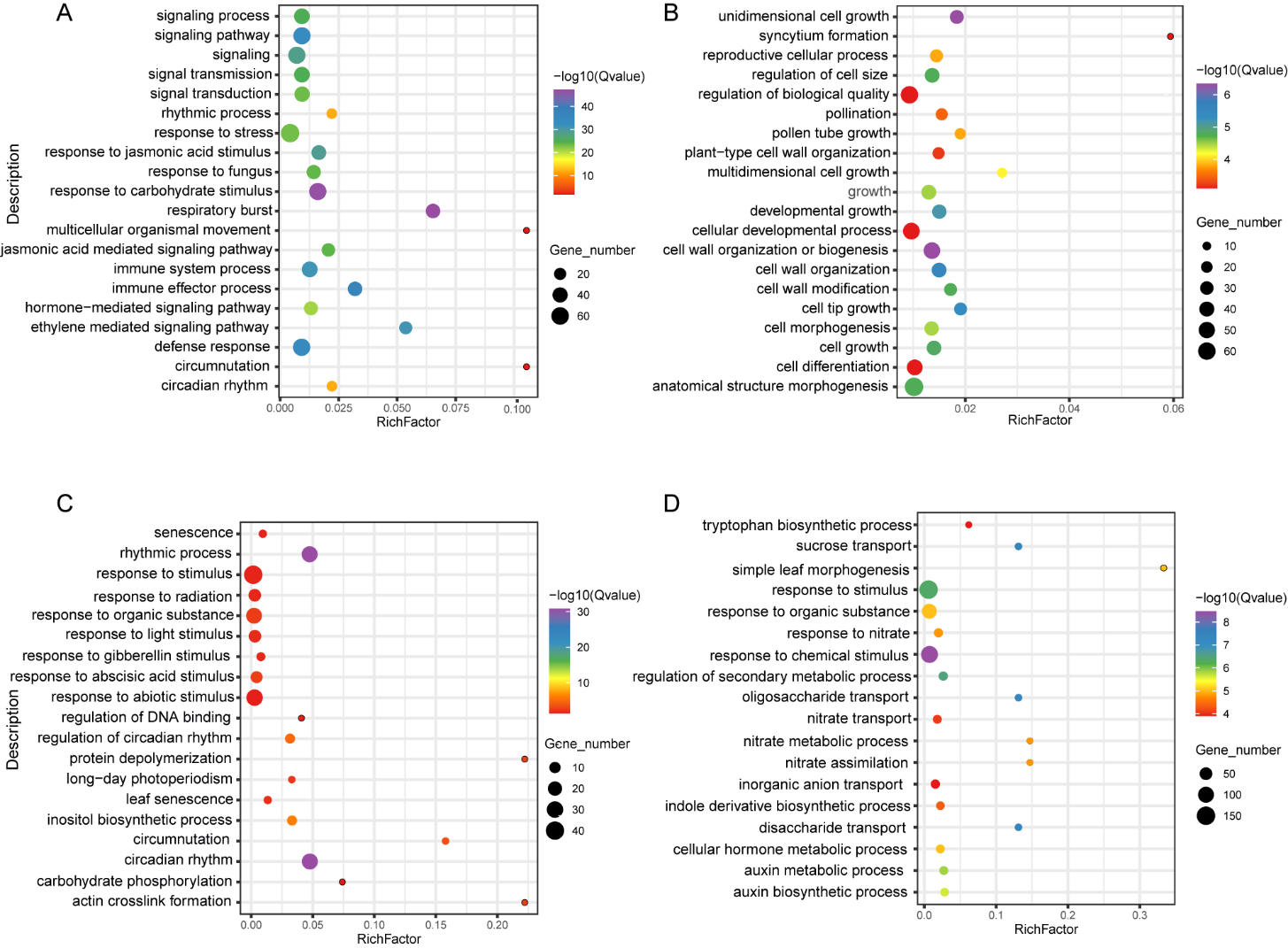


**Supplementary Figure 4.** GO functional classification of the St and Le DEGs from high- and low- HI accessions at CQ and YN. (A) St_CQ_High vs Low. (B) St_YN_High vs Low. (C) Le_CQ_High vs Low. (D) Le_YN_High vs Low.


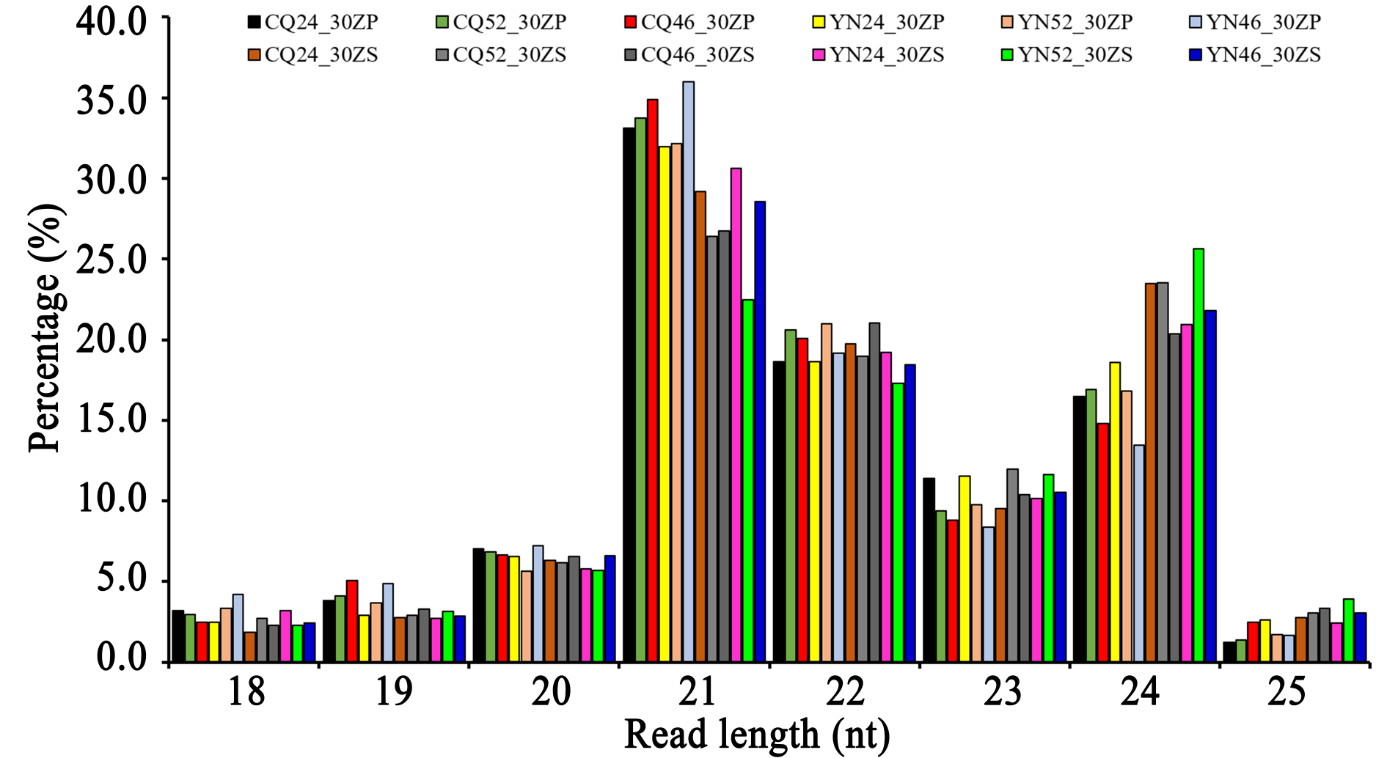


**Supplementary Figure 5.** Size distribution of small RNAs sequenced in this study.


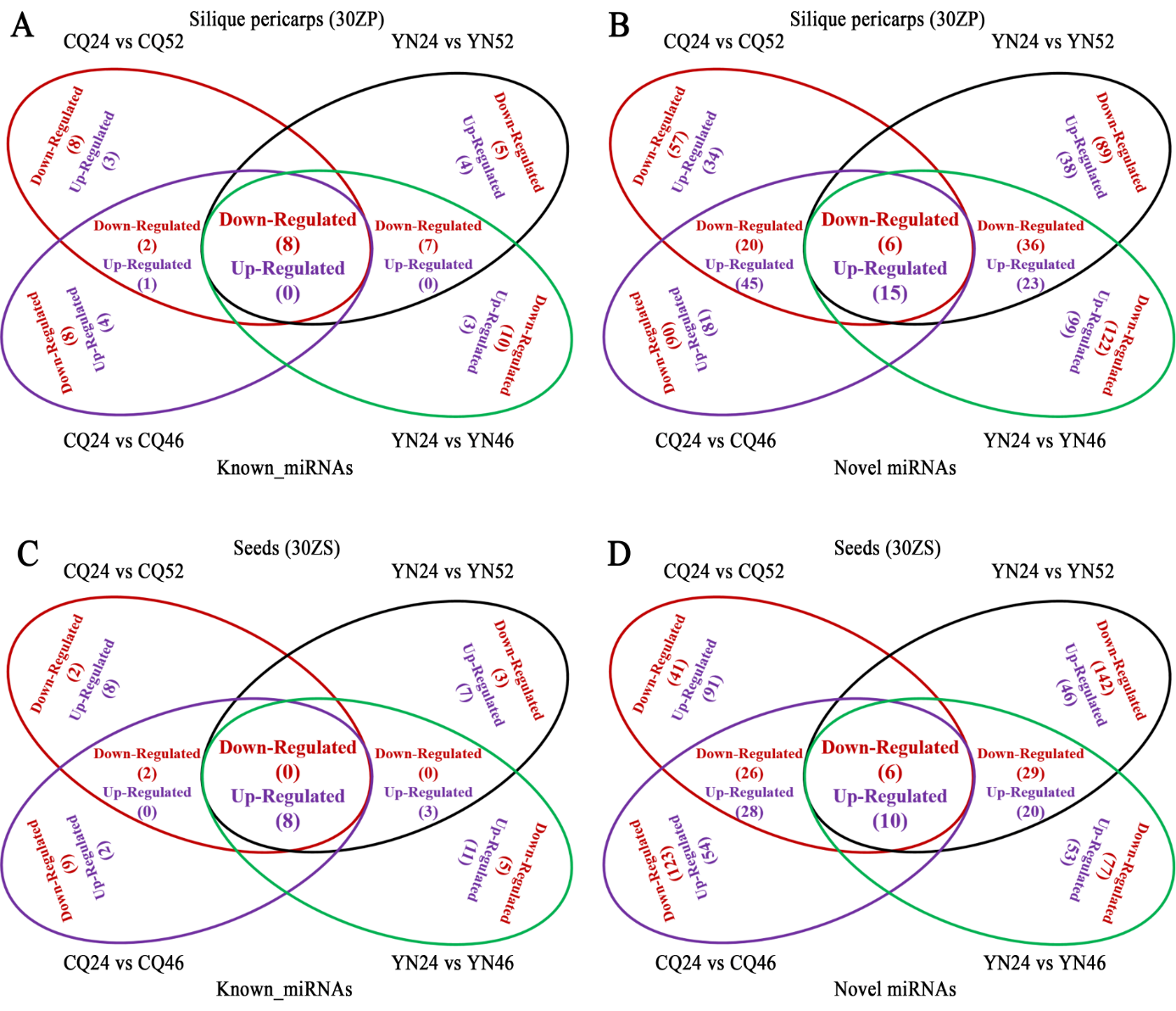


**Supplementary Figure 6.** Venn diagrams showing the number of DEMs.


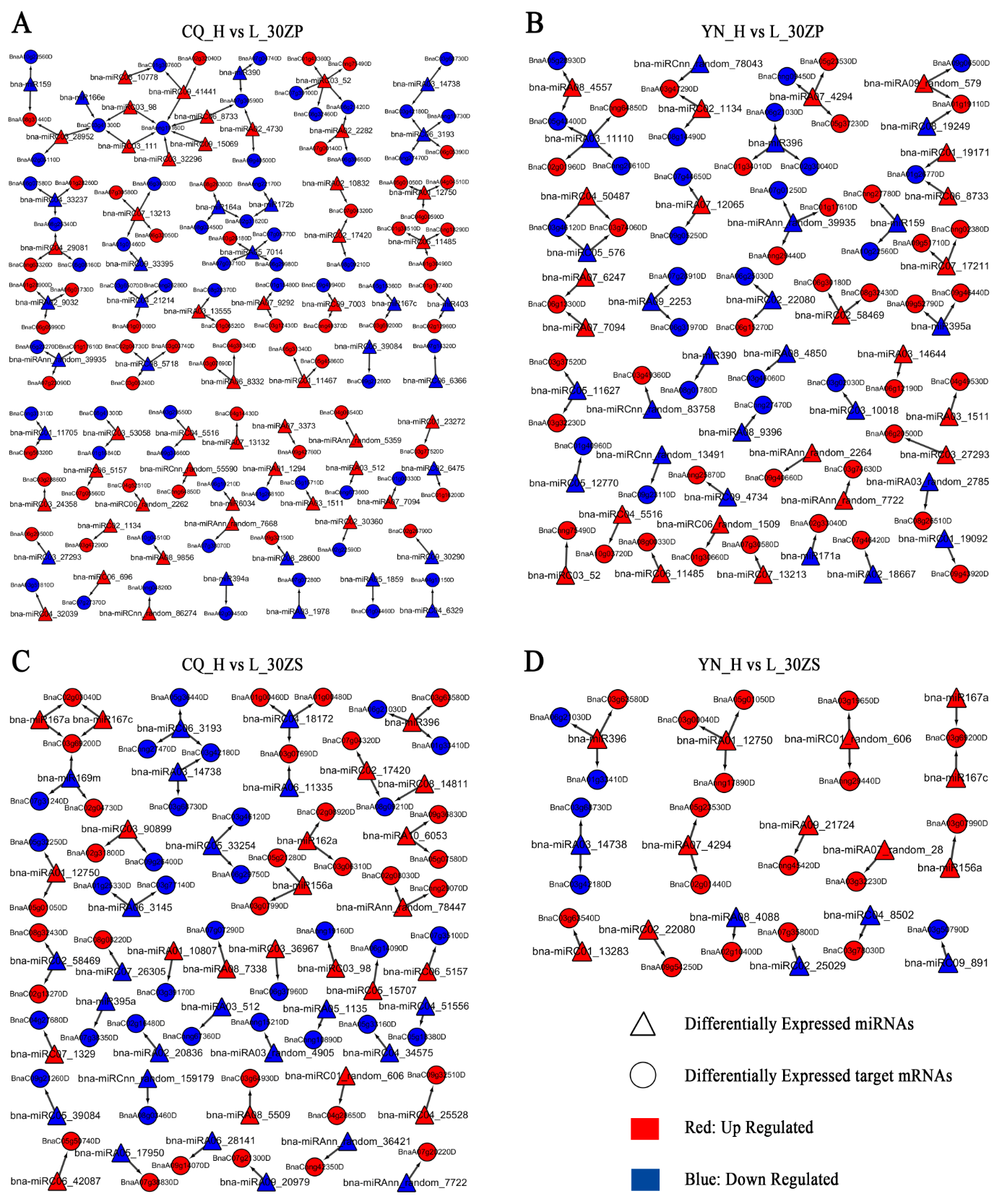


**Supplementary Figure 7.** miRNA-mRNA correlation network in silique pericarps and seeds.


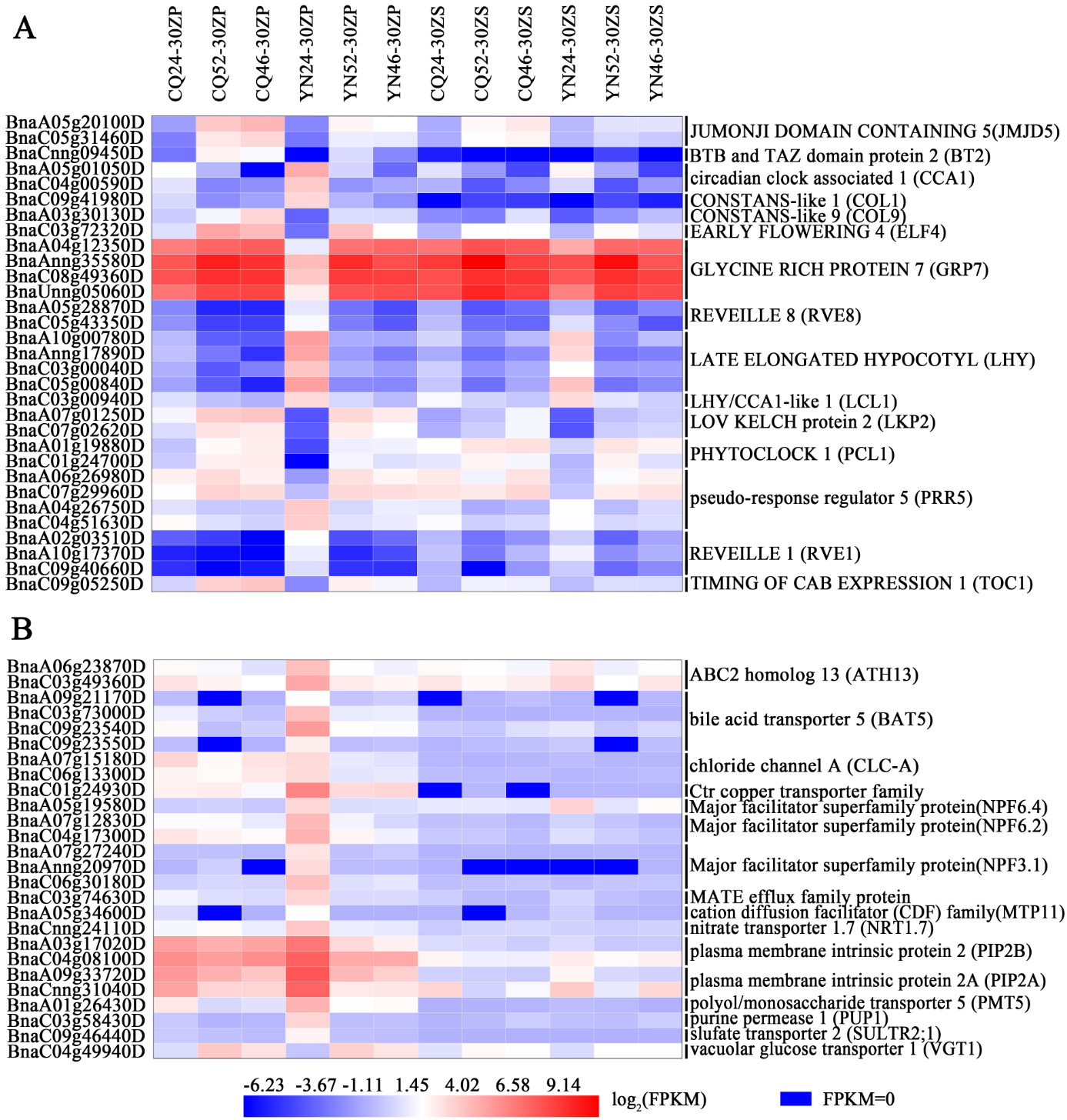


**Supplementary Figure 8.** Heatmap representation of DEGs related to (A) circadian rhythms and (B) transporter activity.

## 2. Supplementary Tables

**Supplementary** **Table 1.** Summary of mRNA sequencing datasets.

**Supplementary** **Table 2.** Lists of differentially expressed genes (DEGs).

**Supplementary** **Table 3.** Traits data of WGCNA at each sampling.

**Supplementary** **Table 4.** Summary of miRNA sequencing datasets.

**Supplementary** **Table 5.** The reads of known and novel miRNAs from sequenced small RNA libraries.

**Supplementary** **Table 6.** Differentially expressed and potential target genes for the DE miRNAs identified in different comparisons.

**Supplementary** **Table 7.** Significantly enriched GO terms for the differentially expressed target genes of the DE miRNAs identified in different comparisons.

**Supplementary** **Table 8.** Primers used for qRT-PCR analysis.
